# Intermediate Autosomal Recessive Osteopetrosis With an Unusual Absence of Fractures

**DOI:** 10.31486/toj.22.0018

**Published:** 2022

**Authors:** Ali Ishaque, Eisha Farid, Sharmeen Nasir, Laila Tul Qadar, Ammarah Jamal

**Affiliations:** ^1^ Department of Internal Medicine, Dow University of Health Sciences, Karachi, Pakistan; ^2^ Department of Pediatrics, Civil Hospital Karachi, Dow University of Health Sciences, Karachi, Pakistan

**Keywords:** *Genetic diseases–inborn*, *osteomyelitis*, *osteopetrosis*

## Abstract

**Background:** Osteopetrosis includes a variety of rare inherited skeletal disorders characterized by increased bone density and thickness. It has different clinical forms, including infantile autosomal recessive, intermediate autosomal recessive, and late-onset autosomal dominant forms. Intermediate autosomal recessive osteopetrosis (IARO) displays high variability.

**Case Report:** A 10-year-old male presented to our pediatrics emergency department with abdominal distension, low-grade fever, and swelling of the right maxilla with associated discharge. His local physician had treated the lesion with drainage and aspiration of pus without improvement. Examination revealed pallor, hepatosplenomegaly, poor dentition, and dental caries. Eye examination showed reduced visual acuity, absent color vision, nystagmus, and bilateral optic nerve atrophy. Laboratory investigations showed anemia and thrombocytopenia. Radiography yielded classic features of osteopetrosis. Detailed intraoral examination revealed an area of exposed necrotic bone in the alveolar region of the right maxilla, leading to a diagnosis of IARO with underlying osteomyelitis. The intraoral wound was treated with bismuth iodoform paraffin paste dressing, and the infection was treated with antibiotics. Anemia and thrombocytopenia were managed supportively by transfusion of packed red blood cells and platelets.

**Conclusion:** IARO commonly presents with multiple fractures, so the absence of fractures in our patient was unusual. Studies evaluating the intermediate variant are meager; hence, documenting its various presentations is essential to aid physicians in making early diagnoses. Osteomyelitis of the jaws is a feared complication in these patients. Therefore, practitioners need to be cautious of infections of dental origin.

## INTRODUCTION

Osteopetrosis, also known as marble bone disease, encompasses a variety of rare hereditary skeletal disorders characterized by increased bone density and thickness secondary to differentiation or functional defects in osteoclasts.^1^ This disorder is classified into different clinical forms, including late-onset autosomal dominant osteope-trosis, infantile autosomal recessive osteopetrosis, and intermediate autosomal recessive osteopetrosis (IARO).^2^ A study conducted in Costa Rica found the incidence of autosomal recessive osteopetrosis to be 3.4:100,000.^3^ Another study conducted in Denmark reported a prevalence of approximately 1:20,000 for the autosomal dominant type.^4^

Patients present with a spectrum of phenotypic expressions, ranging from asymptomatic to rapidly fatal. The most severe manifestations occur in malignant infantile osteopetrosis, a condition discovered at birth or during early infancy.^5^ Most patients with malignant infantile osteopetrosis succumb to infection or hemorrhage in infancy, whereas patients with the autosomal dominant form have a normal life expectancy.^5,6^ The autosomal dominant form presents with characteristic radiographic findings with or without hematologic complications.^5^ IARO displays high variability. It has a milder presentation compared to malignant infantile osteopetrosis, with a range of orthopedic and dental symptoms presenting in childhood.^7^

We report the case of a 10-year-old male who presented to the pediatrics emergency department with complaints of abdominal distension and swelling on his right cheek who was eventually diagnosed with IARO.

## CASE REPORT

A 10-year-old male who had never been vaccinated for any infectious diseases presented to our pediatrics emergency department with progressive abdominal distension for the prior 4 years and swelling of his right cheek for 4 months with associated discharge for 2 months. His primary care physician had treated the lesion with drainage and aspiration of pus. Treatment did not result in improvement, and the lesion began to discharge continuously. These symptoms were associated with a low-grade fever.

The child had had oscillatory eye movements and decreased vision since the first year of life. He had never had any fractures. He had a history of packed red blood cell transfusions at 6 and 8 years, for which no documentation was available. He had delayed developmental milestones. The patient's history was unremarkable for jaundice, hematemesis, melena, epistaxis, gum bleeding, petechiae, easy bruising, or fits. The child was the sixth product of consanguineous marriage. None of his siblings or extended family had a history of similar complaints or transfusions.

**Figure 1.**
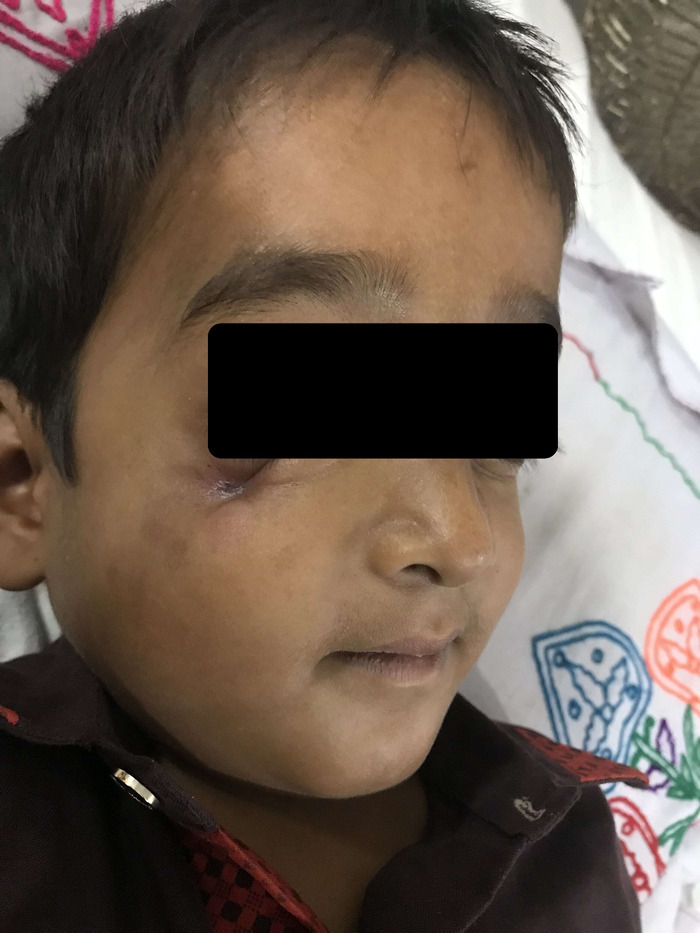
The patient presented with a draining sinus tract on the cheek below the lateral half of the right eye.

On examination, the patient appeared pale with a draining sinus tract on his cheek below the lateral half of the right eye (Figure 1), frontal bossing, bilateral horizontal nystagmus, and poor dentition with carious teeth. The patient was underweight and short for his age, with a weight and height of 20 kg and 118 cm, respectively. His heart rate was 120/min, temperature was 100 °F, blood pressure was 106/64 mm Hg, respiratory rate was 28/min, oxygen saturation was 98% on room air, and capillary refill time was <2 seconds. The child had severe pallor, and a few petechial spots were visible on the lower limbs. Multiple lymph nodes were palpable bilaterally in the submandibular, preauricular, postauricular, and anterior cervical groups. Rachitic rosary was evident. The abdominal examination yielded a soft, distended, nontender abdomen with hepatosplenomegaly. The liver span was 18 cm, and the tip of the spleen was palpable 19 cm below the left costal margin. Detailed eye examination showed an intact visual field with reduced visual acuity and absent color vision. The patient's pupils were equal in size and reactive to light. No afferent pupillary defect was present. The accommodation reflex was normal. The patient had bilateral jerky nystagmus. Fundoscopy revealed bilateral optic nerve atrophy. His hearing was normal. He had no other cranial nerve involvement. The remainder of the physical examination was unremarkable.

Laboratory investigations revealed anemia and thrombocytopenia, with decreased hemoglobin and hematocrit levels. Total leukocyte count was within the normal range. Peripheral blood smear showed normochromic red blood cells with anisocytosis, rouleaux formation, and reactive lymphocytes. Blood smear for malarial parasite was negative. Other laboratory investigations revealed low serum 25-hydroxyvitamin D and calcium. The Table reports the laboratory findings.

**Table.** Laboratory Findings

Variable	Result	Reference Range
Hematologic profile		
Hemoglobin, g/dL	4.7	11.5-13.5
Mean corpuscular volume, fL	90.5	80-95
Hematocrit, %	15.2	30-44
Mean corpuscular hemoglobin, pg	28.0	27-31
Mean corpuscular hemoglobin concentration, g/dL	30.9	33.4-35.5
Total leukocyte count, × 10³/μL	8.1	4.0-11.0
Platelet count, × 10³/μL	17	250-450
C-reactive protein, mg/L	22	<10
Prothrombin time, s	9.3	11-13.5
Activated partial thromboplastin time, s	33.3	30-40
Biochemical profile		
Creatinine, mg/dL	0.2	0.5-1.0
Sodium, mEq/L	131	135-145
Potassium, mEq/L	3.5	3.5-5.0
Chloride, mEq/L	99	96-106
Magnesium, mEq/L	1.8	1.7-2.2
Calcium, mg/dL	8.3	8.5-10.2
Phosphorus, mg/dL	4.2	4.0-7.0
Vitamin D, ng/mL	28.3	>30
Parathyroid hormone, pg/mL	23.9	7-53
Total protein, g/dL	8.1	6.0-8.3
Albumin, g/dL	3.3	3.4-5.4
Globulin, g/dL	4.8	2.0-3.5
Albumin/globulin ratio	0.69	1.1-2.5

**Figure 2.**
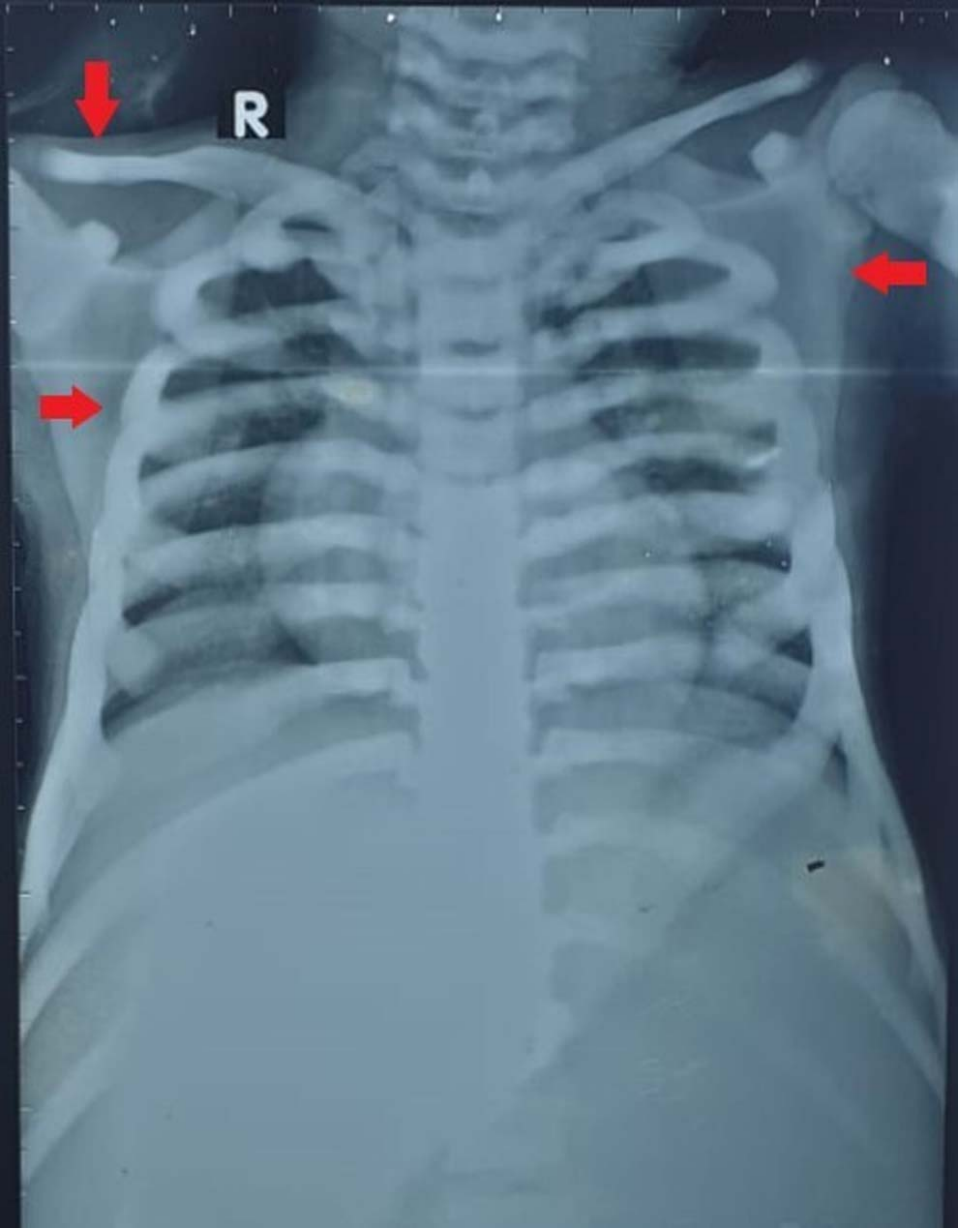
Chest x-ray shows a generalized increase in bone density in the clavicles, scapulae, and ribs (arrows).

**Figure 3.**
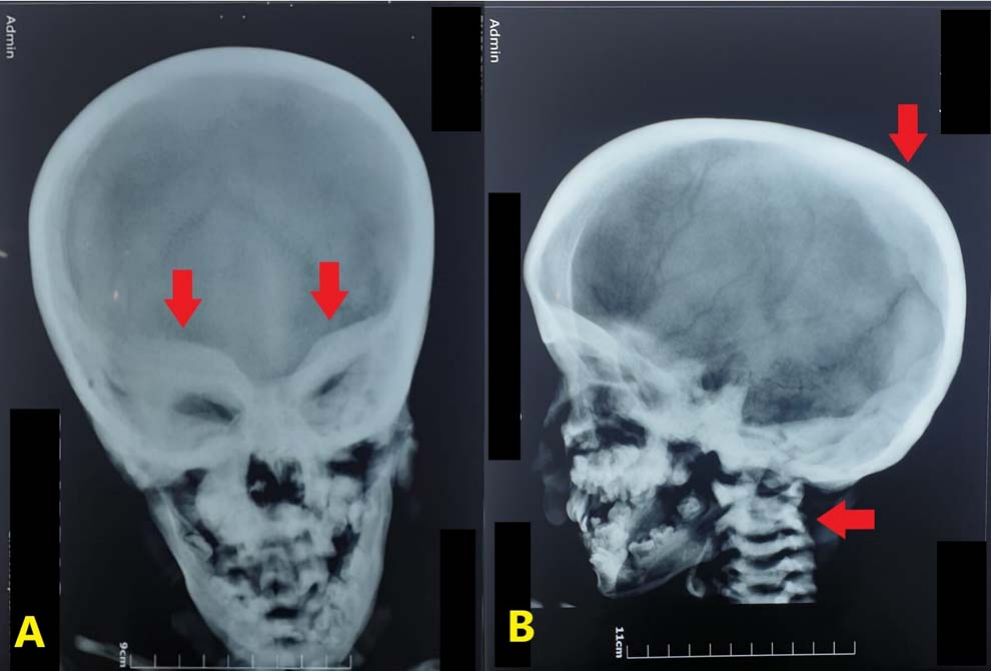
Skull radiographs show (A) periorbital sclerosis (arrows) and (B) thickening of the calvarium and the visualized cervical vertebrae (arrows).

**Figure 4.**
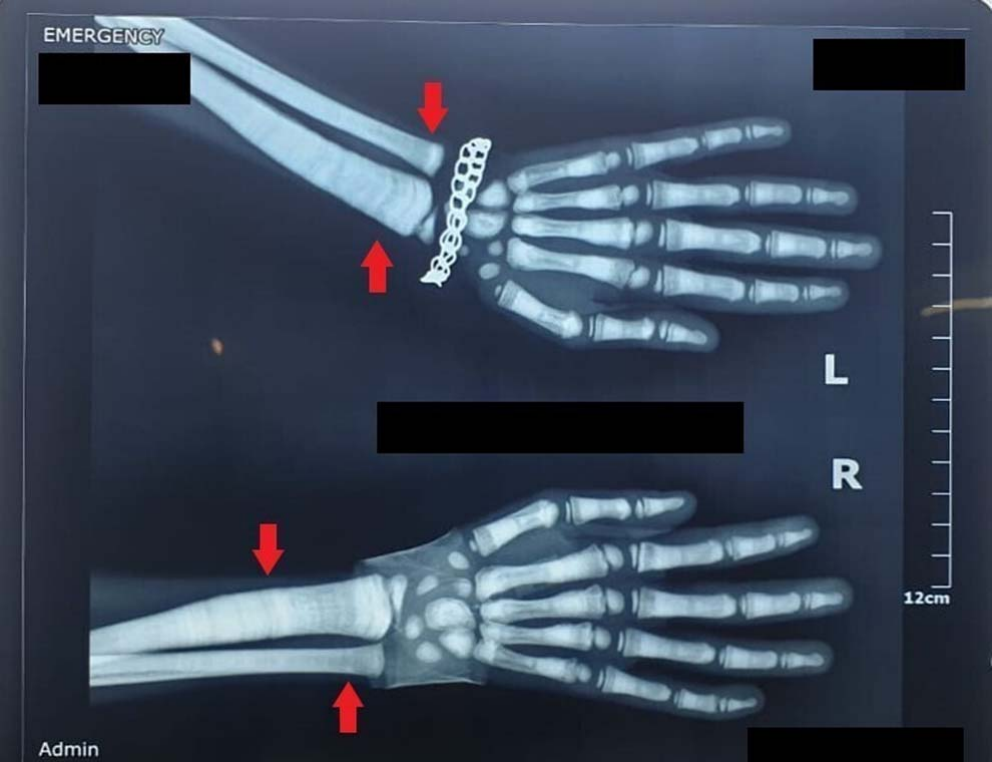
Radiographs of the hands show alternating bands of radiodense and radiolucent lines at the distal metaphyses of the radii and ulnae (arrows).

**Figure 5.**
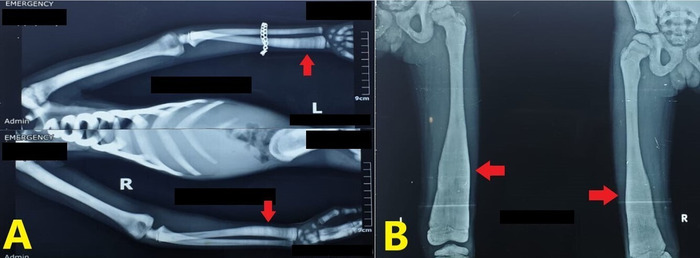
Erlenmeyer flask deformities are seen at the distal ends of (A) the radii (arrows) and (B) the femora (arrows).

**Figure 6.**
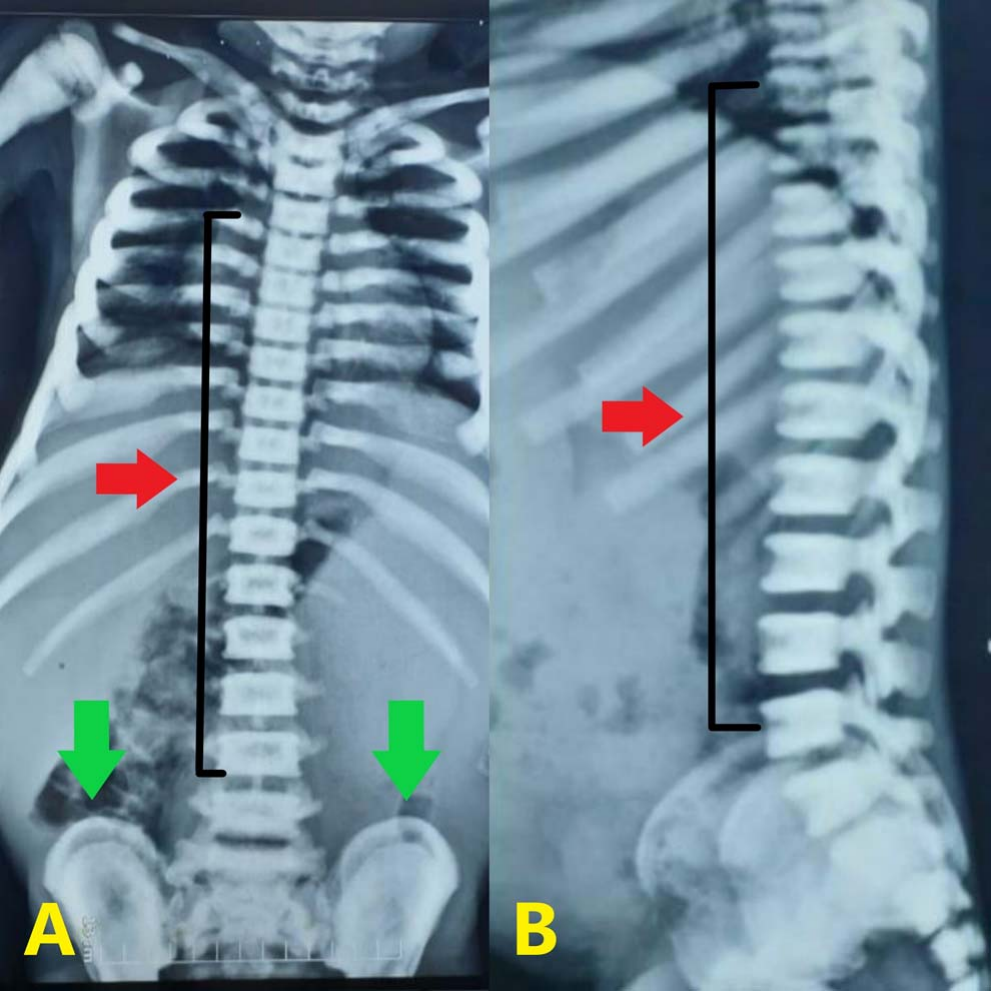
(A) Anteroposterior view of the spine shows a bone-within-a-bone appearance in the iliac blades (lower arrows) and a sandwich vertebrae appearance (upper arrow). (B) Lateral view of the spine shows a sandwich vertebrae appearance (arrow).

Chest x-ray revealed a generalized increase in bone density in the visualized clavicles, scapulae, and ribs (Figure 2). The diffuse osteosclerosis prompted a skeletal survey that showed periorbital sclerosis (Figure 3A). Lateral radiograph of the skull also showed thickening of the calvarium and the visualized cervical vertebrae (Figure 3B). Alternating radiodense and radiolucent lines were seen at the distal metaphyses of the radii and ulnae (Figure 4). However, no evidence of acroosteolysis was seen, which excluded a diagnosis of pyknodysostosis. Erlenmeyer flask deformities, characterized by reduced constriction of the diaphyses, were seen at the distal ends of the radii (Figure 5A) and the femora (Figure 5B). A bone-within-a-bone appearance was visible in the iliac blades, in addition to a sandwich vertebrae appearance because of sclerosis of the vertebral endplates (Figure 6).

A diagnosis of IARO was made based on the clinical and radiologic findings. Imaging showed no evidence of any healed or healing fractures, an otherwise common finding in autosomal recessive and dominant types of osteopetrosis. Concerns that the cutaneous draining sinus may have originated from the patient's dental caries prompted a referral to the oral and maxillofacial department for consultation. Detailed intraoral examination revealed a bony swelling of the maxilla in the upper right quadrant with an area of exposed necrotic bone in the right maxillary alveolar region, consistent with osteomyelitis of the maxilla.

The final diagnosis was IARO with osteomyelitis of the maxilla. The patient was hospitalized and began treatment with irrigation of the intraoral wound with normal saline and application of a bismuth iodoform paraffin paste dressing. Antibiotic therapy was initiated with intravenous (IV) amoxicillin/clavulanic acid 600 mg thrice daily and IV metronidazole 30 mg thrice daily. The purulent discharge decreased 3 days after initiation of therapy. However, 2 days after this transient improvement, discharge from the wound restarted. Pus culture revealed methicillin-resistant *Staphylococcus aureus* sensitive to vancomycin and linezolid. The patient's antibiotics were switched to injection linezolid 10 mg/kg thrice daily for 14 days, after which discharge from the draining sinus tract stopped and the external swelling decreased. The patient was discharged on day 20 with oral linezolid 10 mg/kg thrice daily for 4 more weeks to complete a course of 6 weeks.

During the patient's hospitalization, anemia was managed with 2 transfusions of packed red blood cells 10 mL/kg, once on day 1 and again on day 3, to maintain a hemoglobin level of 8 g/dL. Additionally, 2 units of platelets were transfused on days 1, 2, 6, 7, and 9 to maintain a platelet count >20,000 × 10³/μL.

On discharge, the patient was prescribed oral vitamin D 400 IU once daily and calcium syrup 300 mg thrice daily to be given for 1 month. The parents were counseled regarding the genetic nature of the disease. The possibility of hematopoietic stem cell transplantation (HSCT) as definitive management was explained to them, and they were advised to visit the bone marrow transplant center. The patient was given dental hygiene instructions and advised to follow up monthly in the pediatric outpatient department.

At the patient's first follow-up visit, after completion of 6 weeks of linezolid therapy, he had no sign of external discharge from the draining sinus tract, external swelling had markedly reduced, and the intraoral wound was clean, dry, and healed. Oral linezolid was stopped. Complete blood count, serum calcium, and vitamin D levels were recommended, but the patient was lost to follow-up.

## DISCUSSION

Pathogenesis of recessive forms of osteopetrosis involves mutations of multiple genes leading to impaired osteoclast ruffled border formation necessary for normal bone resorptive activity. These include the CLCN7 (chloride channel 7) and TCIRG1 (T-cell immune regulator 1) genes and the more recently identified PLEKHM1 (pleckstrin homology domain-containing family M member 1) and SNX10 (sorting nexin 10) genes.^8-11^ IARO is highly variable in presentation, with clinical features milder than the malignant form.^7^

At the time of his diagnosis, the patient detailed in our case report was 10 years old. His symptoms started at approximately 6 years of age. He presented with characteristic findings of IARO such as short stature, frontal bossing, and dental caries complicated by osteomyelitis. Patients with the intermediate form often suffer multiple pathologic fractures.^7^ Our patient presented with an unusual absence of fractures, and no signs of healed or healing fractures were visible on radiology. Consistent with our case, Yadav et al reported the absence of fractures in 2 siblings with IARO.^12^

Manifestations of bone marrow failure commonly observed in malignant infantile osteopetrosis, such as hepatosplenomegaly, anemia, and thrombocytopenia with subsequent bleeding, may also be demonstrated in IARO.^5,13^ The progressive invasion of the medullary cavity by the growing bone mass leads to compensatory extramedullary hematopoiesis.^13^ These findings were apparent in our patient.

Some patients may also experience visual and hearing disturbances as a result of cranial nerve compression caused by bone expansion at the cranial nerve foramina. Hearing loss and optic nerve atrophy with progressive visual impairment are the most common manifestations.^14,15^ In severe cases, the vision loss may even progress to complete blindness.^16^ Ocular manifestations in our patient included bilateral optic nerve atrophy, reduced visual acuity, absent color vision, and nystagmus. Nystagmus has been reported previously in a patient with IARO.^12^

Dental problems are a prominent aspect of IARO. In 1954, Bergman and Engfeldt reported dental anomalies including congenitally absent or malformed teeth, delayed tooth eruption, malformed crowns and roots, enamel aplasia, and rampant caries.^17^ The enamel defects and dental caries are secondary to hypocalcification of teeth as a result of diminished hydroxyapatite crystal formation in both the enamel and dentin.^18^ Osteomyelitis of the jaws is a feared complication in all patients. Poor vascularization of the teeth and jaws secondary to constriction of bony canals housing their blood supply and dental caries are important predisposing factors for infection.^19,20^

Localized bone swelling commonly involving the jawbones has been demonstrated in patients with IARO. Mosayebi et al and Xue et al reported swelling of the mandible in their respective patients.^16,21^ Conversely, our patient presented with swelling of the maxilla, subsequently identified as an odontogenic cutaneous draining sinus tract, a lesion on the cutaneous surface of the face or neck that originates in the oral cavity, commonly from dental infections. The infectious debris takes the path of least resistance and breaks through the skin to form draining sinus tracts.^22^ These lesions are frequently misdiagnosed and are often subjected to numerous unsuccessful attempts at incision and drainage before the underlying dental etiology is addressed.^23^ The same was true for our patient, who did not show any improvement following drainage of the lesion by a primary care physician. Thus, medical practitioners must consider dental infections as a possible source of inflammatory facial lesions before attempting a stopgap measure, particularly in the setting of osteopetrosis.

Accurately identifying symptoms and making a diagnosis can prove challenging because of the heterogeneity and highly variable presentation of osteopetrosis. The minimum diagnostic requirement is demonstration of the classic radiologic features of the disease; generalized osteosclerosis, bone-within-a-bone and sandwich vertebrae appearances, Erlenmeyer flask deformities, and alternating radiodense and radiolucent metaphyseal lines visible on standard radiographs. Evidence of healing or healed fractures and skull thickening can also be present.^13^ Our patient exhibited all these features except for signs of new or healed fractures.

Effective management of patients with osteopetrosis requires a multidisciplinary team. All patients should be evaluated by specialists in endocrinology, ophthalmology, genetics, and dentistry following the initial diagnosis. A complete blood count enables the assessment of bone marrow failure and can guide the need for transfusions. Studies such as serum calcium, phosphate, parathyroid hormone, and 25-hydroxyvitamin D should be obtained to monitor mineral homeostasis and determine the need for supplementation. The goal of supplementation is to correct hypocalcemia and maintain vitamin D levels at ≥30 ng/mL.^13^

The only definitive treatment option for autosomal recessive patients is HSCT.^24^ The best results are achieved with genotypically human leukocyte antigen–identical bone marrow donors.^25^ Interferon gamma-1b serves as a bridge to HSCT.^26^ However, HSCT is not without risks and is largely experimental for the intermediate disease.^13,25^

## CONCLUSION

Osteopetrosis is a heterogeneous group of disorders that is highly variable in presentation, particularly the milder IARO. Most patients present with multiple fractures, short stature, vision and hearing loss, and signs of bone marrow failure. Osteomyelitis of the jaws is a common complication, and dental caries are frequently associated. The absence of fractures in our patient was unusual. As studies evaluating the intermediate variant are meager, adding to the existing literature is essential to help physicians and medical practitioners understand the various presentations and make early diagnoses.

## Notes

*This article meets the Accreditation Council for Graduate Medical Education and the American Board of Medical Specialties Maintenance of Certification competencies for Patient Care and Medical Knowledge.*
